# Molecular subtype identification and predictive power of N6-methyladenosine regulator in unexplained recurrent pregnancy loss

**DOI:** 10.3389/fgene.2022.925652

**Published:** 2022-09-02

**Authors:** Jiahui Huo, Qian Chen, Yutong Zhang, Nuo Li, Zhiyu Fu, Ning Ma, Nan Zheng, Nan Cui, Lu Li

**Affiliations:** ^1^ Department of Social Medicine and Health Care Management, School of Public Health, Hebei Medical University, Shijiazhuang, China; ^2^ Hebei Key Laboratory of Environment and Population Health, School of Public Health, Hebei Medical University, Shijiazhuang, China; ^3^ Department of Reproductive Medicine, The First Affiliated Hospital of Xi’an Jiaotong University, Xi’an, China

**Keywords:** recurrent pregnancy loss, N6-methyladenosine, diagnostic model, YTHDF2, immune cell infiltration

## Abstract

The etiology of recurrent pregnancy loss (RPL) is complicated and effective clinical preventive measures are lacking. Identifying biomarkers for RPL has been challenging, and to date, little is known about the role of N6-methyladenosine (m6A) regulators in RPL. Expression data for m6A regulators in 29 patients with RPL and 29 healthy controls were downloaded from the Gene Expression Omnibus (GEO) database. To establish a diagnostic model for unexplained RPL, differential gene expression analysis was conducting for 36 m6A regulators using least absolute shrinkage and selection operator (LASSO) regression. Unsupervised cluster analysis was conducted on hub genes, and probable mechanisms were explored using gene set enrichment analysis (GSEA) and gene ontology (GO) analysis. Correlations between m6A-related differentially expressed genes and immune infiltration were analyzed using single-sample GSEA. A total of 18 m6A regulators showed significant differences in expression in RPL: 10 were upregulated and eight were downregulated. Fifteen m6A regulators were integrated and used to construct a diagnostic model for RPL that had good predictive efficiency and robustness in differentiating RPL from control samples, with an overall area under the curve (AUC) value of 0.994. Crosstalk was identified between 10 hub genes, miRNAs, and transcription factors (TFs). For example, *YTHDF2* was targeted by mir-1-3p and interacted with embryonic development-related TFs such as *FOXA1* and *GATA2*. *YTHDF2* was also positively correlated with *METTL14* (*r* = 0.5983, *p* < 0.001). Two RPL subtypes (Cluster-1 and Cluster-2) with distinct hub gene signatures were identified. GSEA and GO analysis revealed that the differentially expressed genes were mainly associated with immune processes and cell cycle signaling pathway (normalized enrichment score, NES = -1.626, *p* < 0.001). Immune infiltration was significantly higher in Cluster-1 than in Cluster-2 (*p* < 0.01). In conclusion, we demonstrated that m6A modification plays a critical role in RPL. We also developed and validated a diagnostic model for RPL prediction based on m6A regulators. Finally, we identified two distinct RPL subtypes with different biological processes and immune statuses.

## Introduction

Recurrent pregnancy loss (RPL) is a reproductive disorder generally defined as two or more consecutive pregnancy losses before 20–24 weeks (Rai and Regan, 2006). The prevalence of RPL ranges from 1 to 4% in reproductive-aged women and can cause both physical and psychological distress (Dimitriadis et al., 2020). RPL can be attributed to multiple factors, including genetic, endocrine, anatomical, and immunological disorders. However, the causes of RPL have not been fully elucidated and approximately 50% of RPL cases have no known attributable causes (Jaslow et al., 2010). Epigenetic abnormalities were recently reported to be involved in RPL etiopathogenesis (Arias-Sosa et al., 2018). No effective treatment has been identified for RPL, given that the mechanism underlying its occurrence is unknown (Ford and Schust, 2009; Medicine, 2012). However, there is increased focus on trying to identify the molecular networks that are closely associated with RPL in order to develop effective predictive models and interventions. One epigenetic modification, N6-methyladenosine (m6A), is associated with the occurrence and development of multiple female reproductive disorders, including endometriosis and adenomyosis, polycystic ovary syndrome, preeclampsia, and spontaneous miscarriage, and has potential for diagnosis and treatment of RPL (Mu et al., 2022). However, its association with RPL has not been fully determined.

m6A modification is the most common modification in mRNA and non-coding RNA and can affect RNA splicing, translation, and stability, hence impacting a variety of biological processes (Meyer and Jaffrey, 2017). m6A regulators consist of methyltransferases, demethylases, and binding proteins referred to as writers, erasers, and readers, respectively. The writer complex consists of *METTL3/14, WTAP,* and other proteins, with *METTL3* being the core catalytic subunit (Liu et al., 2014). *FTO* and *ALKBH5* are erasers involved in m6A elimination. The m6A-binding proteins act on RNA readers, which usually contain YTH domains, such as *YTHDF1/2/3* and *YTHDC1/2* (Zaccara et al., 2019).

Three studies have investigated the effects of m6A modification on trophoblast function during early pregnancy (Li et al., 2019; Qiu et al., 2021; Xu et al., 2021). *ALKBH5* expression was markedly higher in the chorionic villi of patients with RPL than in the villi of healthy pregnant women, resulting in reduced m6A modification of *CYR61* mRNA and lower stability. *In vitro* experiments also demonstrated that *ALKBH5* inhibits the proliferation and invasive function of trophoblasts at the maternal-fetal interface in patients with RPL, leading to pregnancy failure (Li et al., 2019). Another study found that benzo(a)pyrene diol epoxide, a metabolite of environmental benzo(a)pyrene, upregulated *lncHZ01* expression in trophoblasts. The study showed that *lncHZ01* mediates the upregulation of *MXD1*, promoting *METTL14* transcription and increasing m6A modification and *lncHZ01* stability. This positive feedback loop ultimately inhibits trophoblast proliferation and induces RPL (Xu et al., 2021). Differentially expressed m6A regulators as well as increased m6A modifications have been identified in individuals experiencing spontaneous miscarriages. In particular, *FTO* expression, which is significantly downregulated in chorionic villi and trophoblasts of individuals experiencing spontaneous miscarriages, affects m6A modifications of several genes associated with immune tolerance, immune cell infiltration, and angiogenesis and facilitate the progression of spontaneous miscarriage (Qiu et al., 2021). Although these *in vivo* and *in vitro* studies have elucidated the role of some m6A regulators in miscarriages, they are not comprehensive and have not systematically investigated the regulatory networks of m6A modifications from a multi-omics perspective. In addition, these studies have mainly focused on exploring the mechanisms of action, while the implications for clinical diagnosis and treatment have not been sufficiently studied. Recent studies have revealed that m6A is a potential target for cancer therapy (Huang et al., 2021). Given its key role in female reproductive disorders, m6A-targeted interventions and integrated predictive models of m6A regulators can also be considered potential diagnostic and therapeutic approaches for RPL, although further supporting evidence is required. Several bioinformatics studies on the role of epigenetics in RPL have been published, with many focusing on DNA methylation (Yu et al., 2018) and epigenetic regulation of miRNA (Bahia et al., 2020) and lncRNA (Wang et al., 2017). However, an integrated analysis of m6A modifications in RPL is still lacking.

This study aimed to evaluate the role of m6A modifications in RPL from a multi-omics perspective, to gain insight into the heterogeneity of RPL pathogenesis by identifying RPL molecular subtypes, and to improve clinical diagnosis by establishing a diagnostic model based on m6A regulators. We analyzed the expression of and interaction between m6A regulators in individuals with unexplained RPL using data from the Gene Expression Omnibus (GEO) database. We developed a diagnostic model of RPL with good predictive efficiency and robustness based on 15 m6A regulators screened using least absolute shrinkage and selection operator (LASSO) regression. We then classified RPL samples into two molecular subtypes, assessed differences between them at the immune microenvironment level, and analyzed the direct correlations between hub genes and immune cell infiltration levels.

## Materials and methods

### Data preprocessing

**FIGURE 1:**
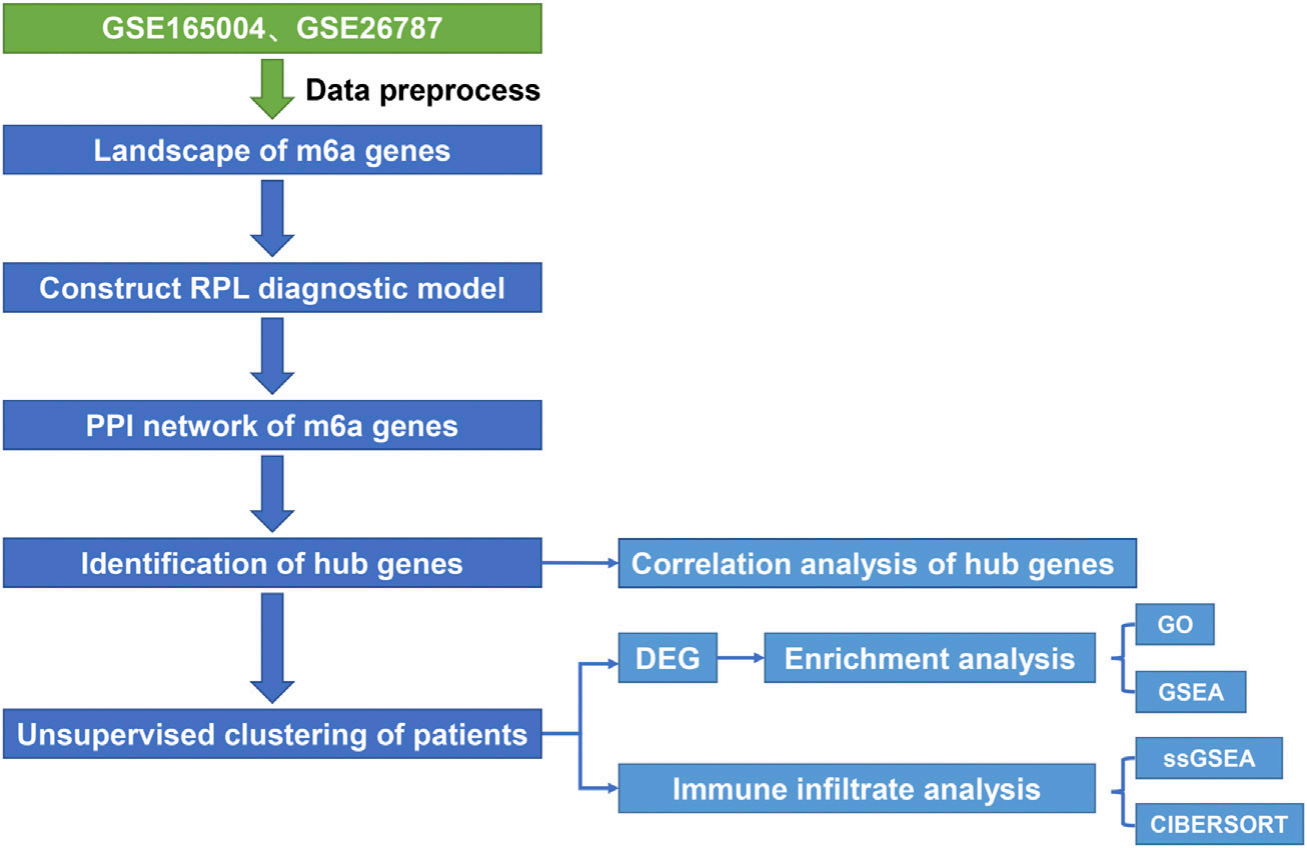
Data preprocessing flow diagram.

The workflow chart (Figure 1) describes the sample sources and analysis strategies at each stage. Microarray data and the correlated clinical information of RPL cases and controls were downloaded from GEO database. All RPL cases with known causes were excluded, and only samples with unexplained RPL causes or unknown risk factors were included in the analysis. Two eligible datasets GSE165004 (https://www.ncbi.nlm.nih.gov/geo/query/acc.cgi?acc=GSE165004, No published literature) and GSE26787 (Lédée et al., 2011) were downloaded, and the sequence platforms were GPL16699 and GPL570 [HG-U133_Plus_2], respectively. GSE165004 contains 48 samples comprising 24 control and 24 RPL samples. GSE26787 contains 10 samples, comprising five control and five RPL samples. All the clinical information used in this study are publicly available in the GEO database. The two datasets were combined for the following analyses. Batch effects correction and log2 normalization were conducted by the “sva” R package (Leek et al., 2012). Box plots were used to visualize the distribution of expression data pre- and post-normalization as well as pre- and post-batch correction.

### Expression landscape of m6A regulators in RPL

A total of 40 known m6A regulators: 11 writers (*METTL3, METTL14, METTL16, RBM15, RBM15B, WTAP, ZCCHC4, PCIF1, CBLL1, ZC3H13 and VIRMA*), 26 readers (*YTHDC1, YTHDC2, YTHDF1, YTHDF2, YTHDF3, IGF2BP1, IGF2BP2, IGF2BP3, HNRNPA2B1, HNRNPC, HNRNPG, FMR1, PRRC2A, eIF3A, eIF3B, eIF3H, LRPPRC, SRSF3, NXF1, TRMT112, NUDT21, CPSF6, SETD2, SRSF10, XRN1* and *ELAVL1*), and three erasers (*ALKBH3, ALKBH5 and FTO*), were obtained from published literature (Chen et al., 2019; Du et al., 2019; Shi et al., 2019; Xu et al., 2020). We overlapped these 40 m6A regulators with available expression profiles from the GEO datasets and obtained 36 common m6A regulators. These 36 m6A regulators included 9 writers (*METTL3, METTL14, RBM15, RBM15B, WTAP, ZCCHC4, PCIF1, CBLL1 and ZC3H13*), 24 readers (*YTHDC1, YTHDC2, YTHDF1, YTHDF2, YTHDF3, IGF2BP1, IGF2BP2, IGF2BP3, HNRNPA2B1, HNRNPC, FMR1, PRRC2A, eIF3A, eIF3B, eIF3H, LRPPRC, SRSF3, NXF1, TRMT112, NUDT21, CPSF6, SETD2, SRSF10* and *XRN1*) and three erasers (*ALKBH3, ALKBH5* and *FTO*).

We generated a heatmap using the R package “pheatmap” (Kolde, 2018) and a grouped box plot using the R package “ggpubr” (Kassambara, 2020) based on the RPL and control group data. Differences in the expression of the 36 m6A regulators in the two groups were analyzed using the Wilcoxon rank-sum test, with statistical significance set at *p* < 0.05. A Circos plot of the 36 genes was generated using the R package “RCircos” (Zhang et al., 2013), with chromosomal location information obtained from the ENSEMBL database (Yates et al., 2020).

### Analysis of correlations between m6A regulators

Correlations between the expression levels of writers and erasers in all patients were measured using Pearson’s correlation coefficient analysis. Absolute correlation coefficient values higher than 0.4 and *p* < 0.05 were considered significant. The R package “ggplot2” was used to generate scatter plots and correlation coefficient curves for gene pairs that met these criteria (Villanueva and Chen, 2019), and histograms were generated using the R package “ggExtra” (Attali and Baker, 2019).

To analyze correlations between hub genes, heatmaps were generated using the R package “Corrplot” (Wei, 2017). Bubble plots were used to visualize correlations between hub genes. Scatter plots and coefficient curves of the most significantly correlated gene pairs were generated as previously described.

### Construction of a diagnostic model based on m6A regulators

Least Absolute Shrinkage and Selection Operator (LASSO) regression was carried out to screen for RPL-associated m6A regulators using the R package “glmnet” (Friedman et al., 2021) and the optimal lambda value was selected. Only genes with non-zero coefficients were retained. The genes and corresponding coefficients used for diagnostic model construction were visualized using a forest plot generated using the R package “forestplot” (Gordon and Lumley, 2019). The risk score was generated using the following formula: risk score = Expression_mRNA1_ × Coefficient_mRNA1_+ Expression_mRNA2_ × Coefficient_mRNA2_ +. . .Expression_mRNAn_× Coefficient_mRNAn_.

The R package “rms” (Harrell, 2018) was used to run a logistic regression model based on the top four absolute weight genes in the LASSO model and the output was visualized using a nomogram. To validate the predictive efficiency of the diagnostic model, receiver operating characteristic (ROC) curves of induvial genes were generated using the R package “pROC” (Robin et al., 2011) and the area under the curve (AUC) was calculated. The closer the AUC is to 1, the better the prediction performance. Internal datasets and decision curve analysis (DCA) was used to illustrate the validity of the nomogram. The DCA curve was plotted using the R package “ggDCA” (Fitzgerald et al., 2015).

### Protein-protein interaction (PPI) networks of m6A regulators

A PPI network of 36 m6A regulators was generated using the STRING database (https://string-db.org/) (von Mering et al., 2003), with a default threshold of 0.4. Cytoscape (Shannon et al., 2003) was used to calculate the network attributes of each node, and Cytohubba (Chin et al., 2014) was used for hub node mining based on the degree of the nodes. The top 10 nodes with the highest degree were defined as hub nodes or hub genes, which have a high level of connection with other nodes. The hub genes may play an extremely important role in the regulation of the entire biological process, and are worthy of further study.

As special gene regulatory elements, miRNAs and transcription factors (TFs) are of great significance to the function of protein-coding genes, and they can also indirectly reflect the functional connections and differences of the genes themselves. For further prediction studies, the miRNAs and TFs of 10 hub genes were predicted using the miRNet database (Chang et al., 2020). Cytoscape was used for data processing and visualization.

### Unsupervised cluster analysis

Unsupervised cluster analysis was conducted using the R package “ConsensusClusterPlus” with cycle computation 1,000 times to ensure stability and reliability (Wilkerson et al., 2013). Based on the expression of the 10 hub genes, the RPL patients were classified into two clusters using the optimal k-means clustering (“kmeans” function in R). Correlations between the expression levels of the 10 hub genes in the two clusters were analyzed and the results were presented on a heatmap. A grouped violin plot of 10 hub genes mRNA expression in two clusters was generated using the R package “ggpubr” (Kassambara, 2020). Groups were compared using Wilcoxon rank-sum test and *p* < 0.05 was considered statistically significant.

### Gene set enrichment analysis (GSEA)

Differential expression was analyzed, with cut-off values set at *p* < 0.05 (adjusted) and log_2_FC > 0.5. Differentially expressed genes (DEGs) were subjected to GSEA, a computation method used to determine the statistical significance of a set of DEGs between two biological states and is commonly used to estimate changes in pathways and biological processes (Subramanian et al., 2005). Gene Ontology (GO) enrichment analysis is commonly used to investigate the large-scale functional enrichment of genes in different dimensions and levels, generally at three levels: biological process (BP), molecular function (MF), and cellular component (CC). To identify highly enriched biological processes, GO functional annotations were performed on all DEGs using the R package “clusterprofiler” (Yu et al., 2012; Wu et al., 2021). Enriched results (*p* < 0.05) were visualized using the R package “GOplot” (Walter et al., 2015). To explore differences in biological processes between the two sets of samples, the gene sets “c5. go. v7.4. entrez. Gmt” and “c2. cp. kegg. v7.4. entrez. Gmt” were downloaded from the MSigDB database (Liberzon et al., 2015). GSEA, implemented in the R package “clusterProfiler”, was used for enrichment analysis and visualization. *p* < 0.05 was considered statistically significant.

### Analysis of immune infiltration

The immune microenvironment is mainly composed of immune cells, inflammatory cells, fibroblasts, interstitial tissues, and various cytokines and chemokines. The analysis of immune infiltration could guide disease therapy and prognosis prediction. ssGSEA, implemented using the R package “GSVA” (Hänzelmann et al., 2013), was used to explore similarities and differences in immune cell infiltration levels between the two groups. Marker genes of 28 immune cells were obtained from literature (Charoentong et al., 2017) and used as background gene sets for ssGSEA. Immune cell infiltration was visualized using a heatmap and a boxplot. Correlations between immune cell infiltration in different disease states in the two groups were visualized using correlation plots generated using the R package “corrplot” (Steen et al., 2020). The R package “CIBERSORT” was used to evaluate immune cell infiltration levels to verify the accuracy of the results. The calculations were based on the LM22 background gene set included with the ‘CIBERSORT’ package. Correlation scatter plots and curves were generated for the hub gene-immune cell pairs as previously described.

### Statistical analysis

All data calculations and statistical analyses were performed using the R software (version 4.1). The predictive efficiency of the diagnostic model was evaluated by ROC curves and the area under the curve (AUC) values. Internal datasets and decision curve analysis (DCA) was used to illustrate the validity of the nomogram. For the comparison of two groups of independent variables, the differences between non-normally distributed variables were analyzed by Wilcoxon rank sum test. All statistical *p* values were bilateral, and *p* < 0.05 was considered statistically significant.

## Results

### Expression landscape of m6A regulators in RPL

To construct a landscape of m6A regulators in RPL, the expression profiles of GSE165004 and GSE26787 datasets from the GEO database were integrated (Supplementary Table S1). Because datasets from different sources generally show strong batch effects, we first examined the distribution of gene expression in the original data before and after batch effect correction. As shown in Supplementary Figure S1, the samples showed strong batch effects when they were integrated, exhibiting considerably different expression distribution characteristics. After batch effect correction and log standardization, the overall expression distribution of all samples converged, improving the accuracy and robustness of subsequent analysis.

**FIGURE 2:**
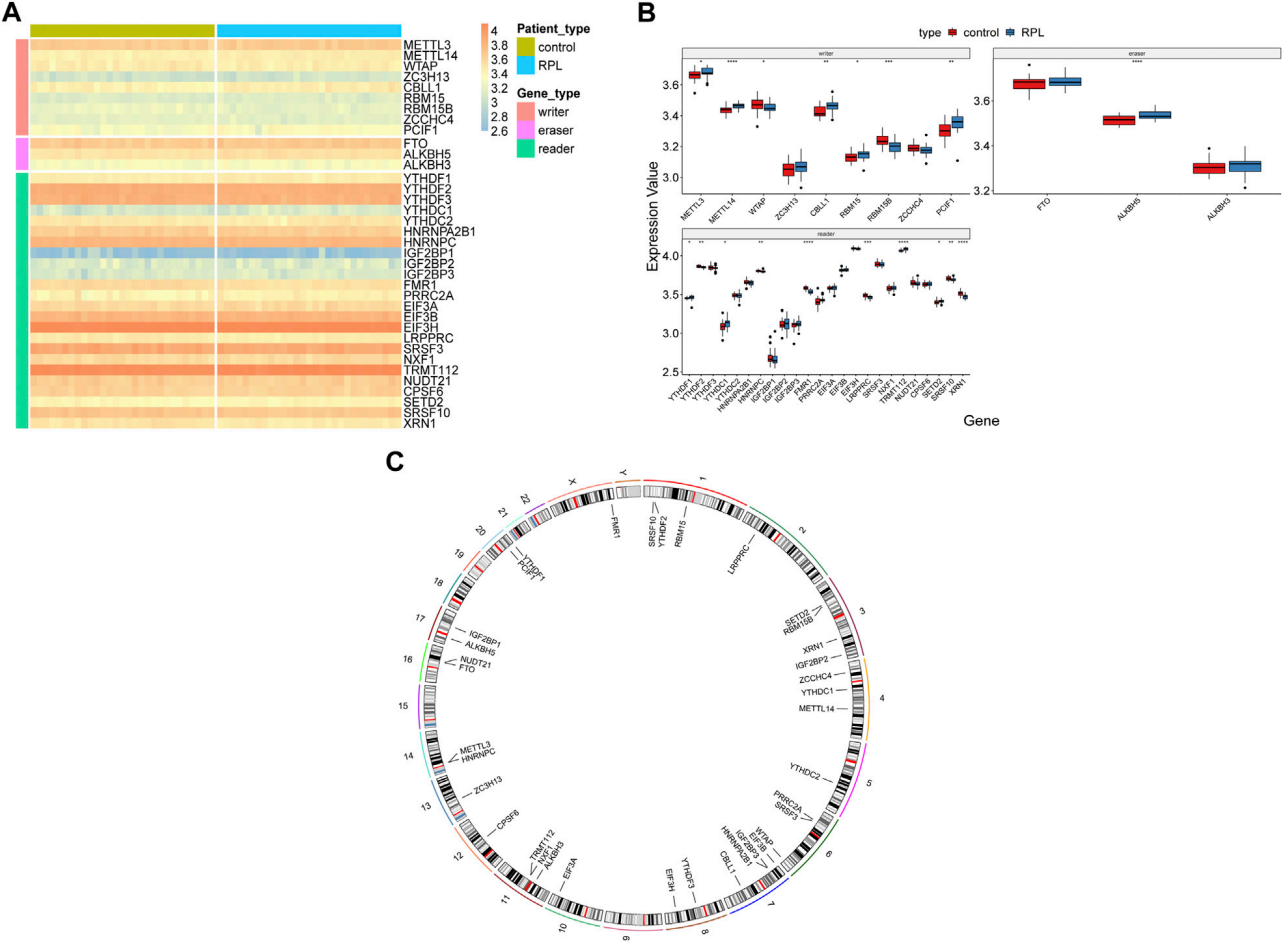
Expression landscape of m6A regulators in Recurrent Pregnancy Loss. **(A,B)** Heatmap and box plot showing differences in the expression levels of 36 m6A regulators in RPL and control samples. Wilcoxon rank-sum test was used to measure statistical differences. **(C)** Circos plot indicating the chromosomal location of the 36 m6A regulators. *p < 0.05; **p < 0.01; ***p < 0.001; ****p < 0.0001; NS represent no significant differences. m6A, N6-methyladenosine, RPL, recurrent pregnancy loss.

The samples were divided into RPL (29 samples) and control (29 samples) groups, and differential expression of the 36 m6A regulators (9 writers, three erasers, and 24 readers) between the groups was analyzed (Figure 2A). A total of 18 m6A regulators showed significant differences in expression in RPL samples, of which 10 were upregulated and eight were downregulated (Figure 2B, *p* < 0.05). The upregulated genes included five writers (*METTL3, METTL14, CBLL1, RBM15* and *PCIF1*), one eraser (*ALKBH5*), and four readers (*YTHDF1, YTHDC1, TRMT112* and *SETD2*). The downregulated genes included two writers (*WTAP* and *RBM15B*) and six readers (*YTHDF2, HNRNPC, FMR1, LRPPRC, SRSF10* and *XRN1*). Additionally, we analyzed the chromosomal location of the 36 m6A regulators and generated a chromosome location landscape (Figure 2C). We found that some regulators were located close to each other, for example, *YTHDF2* and *SRSF10* on chromosome 1, *RBM15B* and *SETD2* on chromosome 3, and *METTL3* and *HNRNPC* on chromosome 14, indicating that they were closely correlated at the genomic level and might have similar expression characteristics at the transcriptomic level.

### Analysis of correlations between writers and erasers in RPL

**FIGURE 3:**
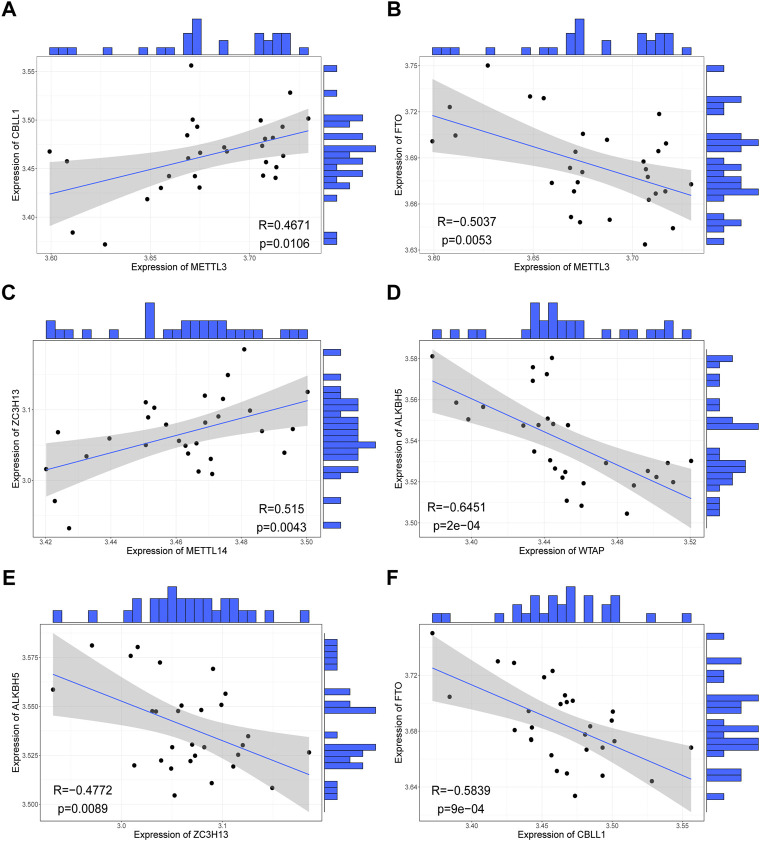
Correlations between expression levels of writers and erasers in all samples. **(A)** *METTL3-CBLL1* correlation. **(B)** *METTL3-FTO* correlation. **(C)** *METTL14-ZC3H13* correlation. **(D)** *WTAP-ALKBH5* correlation. **(E)** *ZC3H13-ALKBH5* correlation. **(F)** *CBLL1-FTO* correlation.

We investigated correlations between the expression levels of writers and erasers in the RPL samples and found highly consistent correlations between the writers and erasers. Overall, the expression of erasers and writers was negatively correlated, consistent with the fact that they regulate contrasting biological functions (Figure 3). For instance, *METTL3-FTO* (*r* = -0.50, *p* < 0.01, Figure 3B), *WTAP-ALKBH5* (*r* = -0.65, *p* < 0.001, Figure 3D), *ZC3H13-ALKBH5* (*r* = -0.48, *p* < 0.01, Figure 3E), and *CBLL1-FTO* (*r* = -0.58, *p* < 0.001, Figure 3F) were negatively correlated in RPL samples, while *METTL3-CBLL1* (*r* = 0.47, *p* < 0.05, Figure 3A) and *METTL14-ZC3H13* (*r* = 0.52, *p* < 0.01, Figure 3C) were positively correlated in RPL samples. Collectively, these results suggest that m6A regulators are involved in the occurrence of RPL and that crosstalk between writers and erasers may play an important role in RPL.

### Construction of an RPL diagnostic model based on m6A regulators

**FIGURE 4:**
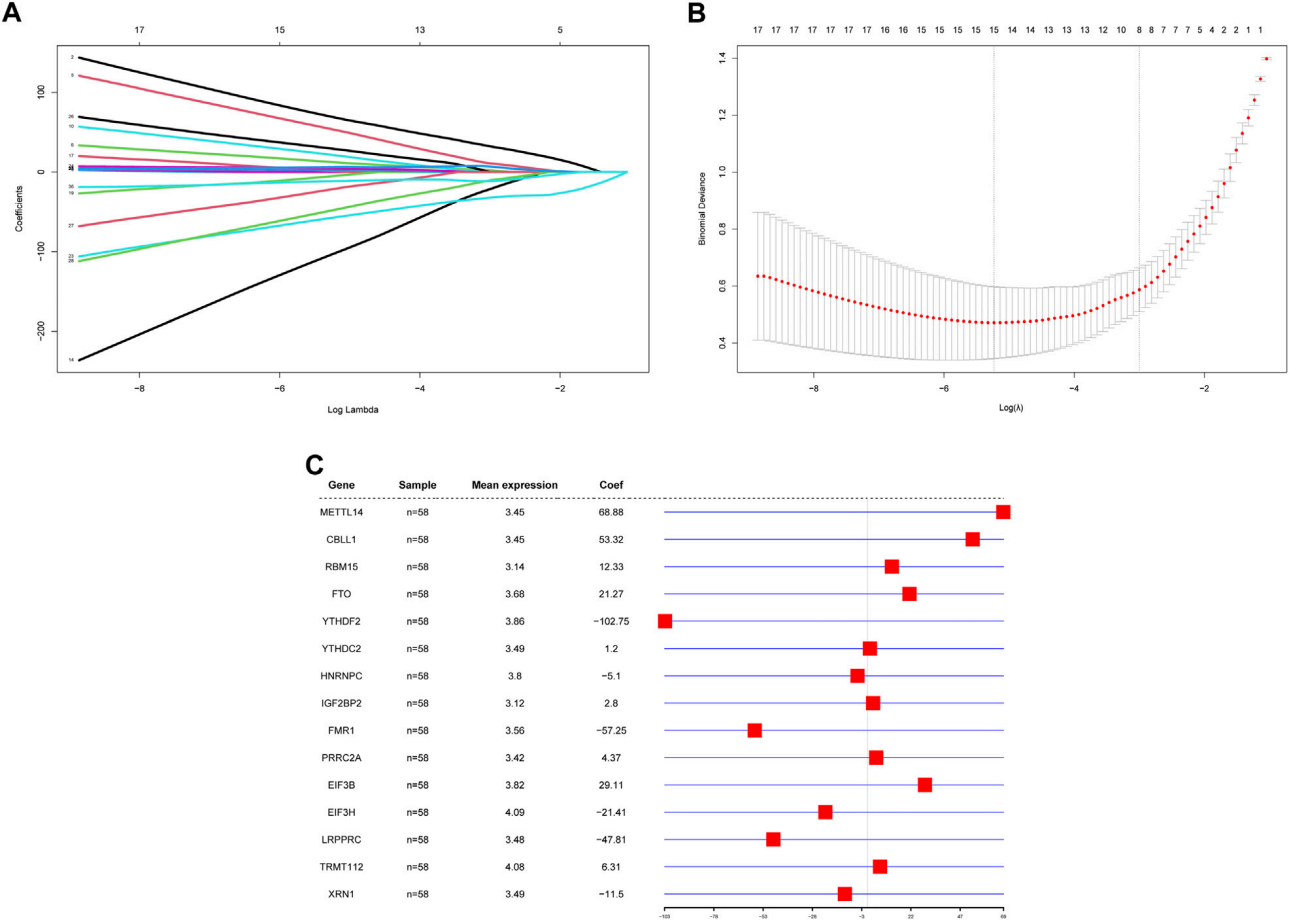
Construction of a diagnostic model based on m6A regulators. **(A)** LASSO regression curve. Shrinkage and selection process for the 36 m6A regulator features using LASSO regression. **(B)** lambda value selection curve. The partial likelihood deviance is plotted against log (λ), where λ is the tuning parameter. Partial likelihood deviance values are shown, with error bars representing standard errors. The optimal lambda value is shown on the left dashed line. **(C)** Diagnostic model forest plot. The first column contains the 15 genes that make up the model, the second column contains the number of samples, the third column contains the mean expression values of these genes, and the fourth column and corresponding graphs contain the effect coefficients of these genes in the model. LASSO: Least absolute shrinkage and selection operator.

Because of the critical role of the m6A modification process, RPL and control samples may have different m6A modification statuses, making it feasible to construct diagnostic models based on m6A regulators. To investigate the contribution of m6A regulators to RPL occurrence, we constructed a diagnostic model of RPL based on all m6A regulators. The 36 m6A regulators were screened for feature selection using LASSO regression, and the optimal lambda value was determined. After screening, a total of 21 nonessential regulators were excluded, with 15 m6A regulators being found to be essential for RPL: *METTL14, CBLL1, RBM15, FTO, YTHDF2, YTHDC2, HNRNPC, IGF2BP2, FMR1, PRRC2A, EIF3B, EIF3H, LRPPRC, TRMT112* and *XRN1* (Figures 4A,B). Subsequently, a diagnostic model consisting of these 15 m6A regulators was constructed to distinguish between RPL and control samples based on risk scores (Figure 4C). Risk scores for the 15 m6A regulators were higher in the RPL group than in the control group. The four regulators with the highest absolute influence coefficient values were *YTHDF2* (-102.75), *METTL14* (68.88), *FMR1* (-57.25), and *CBLL1* (53.32).

**FIGURE 5:**
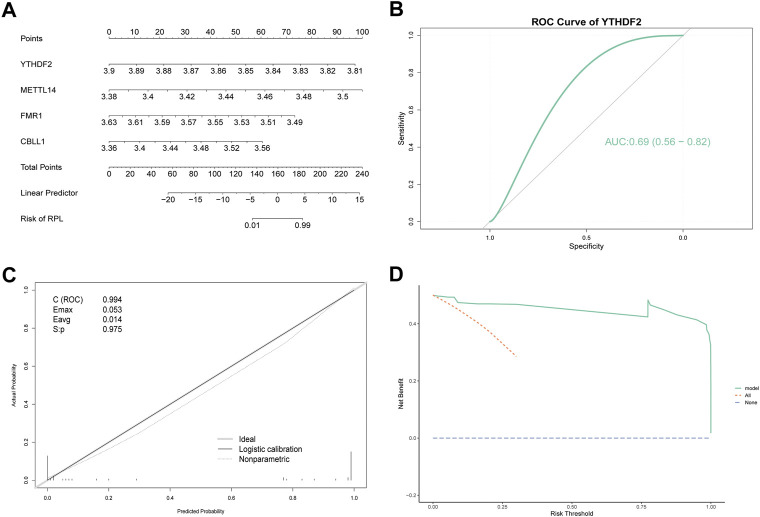
Verification of a diagnostic model based on m6A regulators. **(A)** Nomogram showing the predicted risk of RPL. Predictors are shown on the left and scales are shown on the right. **(B)** ROC curve of *YTHDF2*. *YTHDF2* had the largest absolute coefficient in the diagnostic model. **(C)** Recall Curve. E_max_ is the maximum offset of the model from the ideal model, and E_avg_ is the minimum offset of the model from the ideal model. C (ROC) represents the area under the ROC curve. S: *p* > 0.05 indicates calibration test pass. **(D)** DCA Curve. The purple dashed line represents 0 net benefit rate, all (orange dashed line) indicates that all samples received the intervention, and model (green solid line) represents the model curve. RPL, recurrent pregnancy loss, DCA, decision curve analysis, ROC, receiver operator characteristics.

To validate the accuracy of the diagnostic model, we used the top four regulators (*YTHDF2, METTL14, FMR1* and *CBLL1*) to construct a logistic multi-factor model, and visualized the results using a nomogram, which can quickly help one identify linear predictors and RPL risks (Figure 5A). These four regulators had a large influence on the diagnostic model, indicating the superior accuracy of this diagnostic model. Subsequently, the recall curve, ROC curve of *YTHDF2*, and DCA curve were used to further validate the predictive efficacy of the model. The recall curve showed that the model had an overall AUC of 0.994, with excellent diagnostic prediction power. The area under the ROC curve value of *YTHDF2*, the regulator with the largest absolute coefficient, was 0.69 (Figure 5B). The maximal excursion (E_max_ = 0.053) and average excursion (E_avg_ = 0.014) values were relatively small, indicating that the model was closer to the ideal model. The model also passed the calibration test (S: *p* = 0.975 > 0.05) (Figure 5C). The DCA curve showed that the model yielded additional benefits over all interveners and non-interveners, indicating a good clinical effect (Figure 5D). Overall, the diagnostic model showed outstanding predictive performance and robustness using all three validation approaches. Thus, this diagnostic model can effectively classify individuals into RPL or healthy control groups.

### PPI network of m6A regulators

**FIGURE 6:**
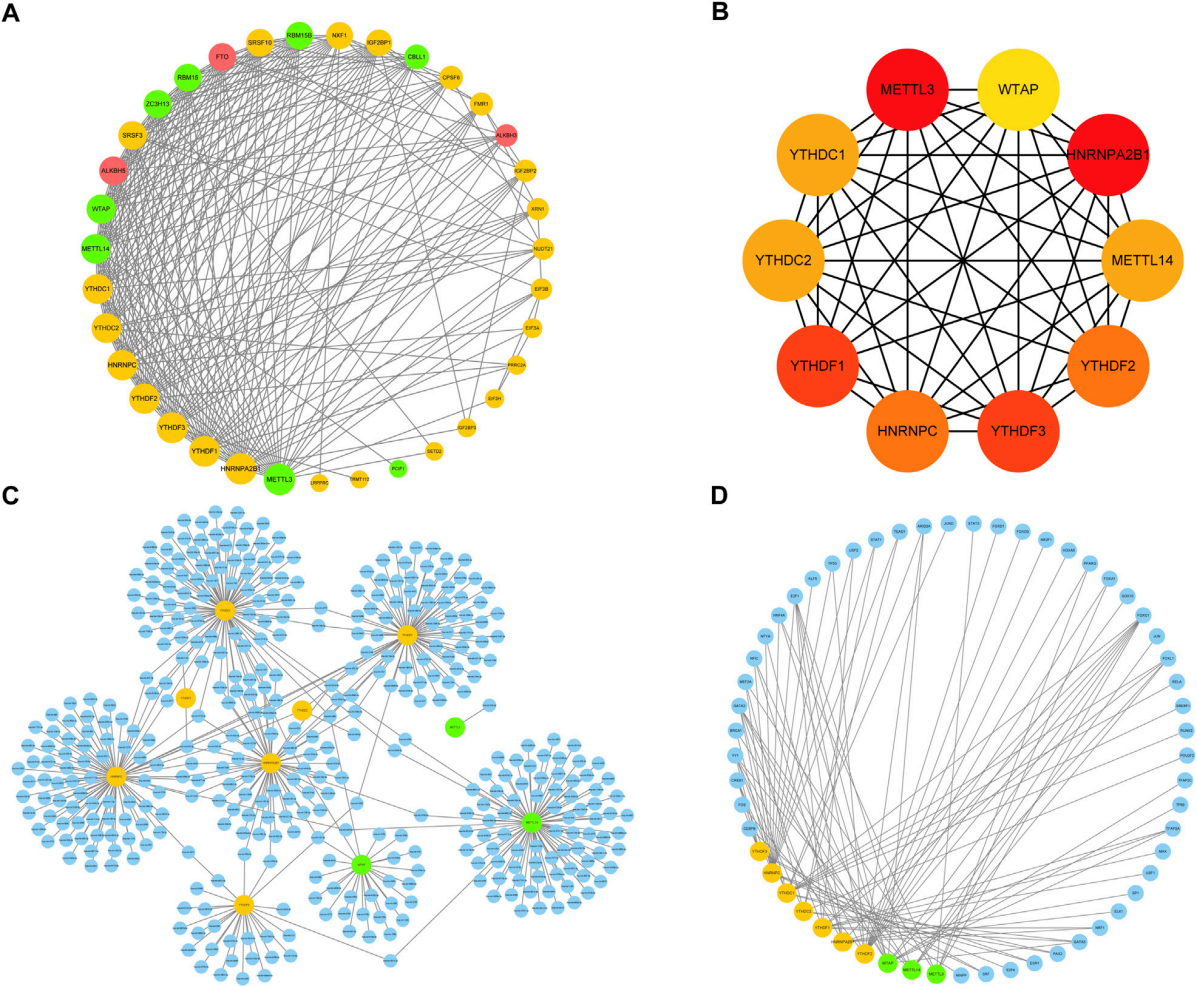
Protein–protein interaction (PPI) network. **(A)** PPI network of 36 m6A regulators. Yellow nodes represent readers, green nodes represent writers, and red nodes represent erasers; the larger the size of the node, the higher the degree of the connection. **(B)**. A sub-network of 10 hub genes extracted from the PPI network was constructed; the deeper the color of the node, the greater the degree of the node in the original network. **(C)** miRNA prediction network of hub genes. Blue nodes represent miRNAs, yellow nodes represent readers, and green nodes represent writers. **(D)** Transcription factor prediction network of hub genes. Blue nodes represent transcription factors, yellow nodes represent readers, and green nodes represent writers. PPI, protein-protein interaction; m6A, N6-methyladenosine.

An analysis of the PPI network was performed to further investigate interactions between the m6A regulators (Figure 6A). The results showed that the 36 m6A regulators interacted closely, indicating that writers, erasers, and readers did not function in isolation, but rather collaborated, during RPL. A few regulators revealed high degrees of connection to others, and the top 10 highly associated genes: *METTL3, HNRNPA2B1, YTHDF1, YTHDF3, YTHDF2, HNRNPC, YTHDC1, YTHDC2, METTL14*, and *WTAP*, were selected as hub genes for further analysis (Figure 6B). These 10 hub genes may play crucial roles in regulating the biological mechanisms underlying RPL.

Next, we investigated the regulatory networks and genetic backgrounds of the hub genes. We used the miRNet database to predict miRNAs and TFs regulated by these hub genes. *YTHDC1, YTHDF1/3, HNRNPA2B1, HNRNPC, METTL14,* and *WTAP* were identified as predicted targets of numerous miRNAs, whereas relatively few miRNAs targeted *YTHDC2, YTHDF2,* and *METTL3*, although they could form miRNA regulatory networks directly or indirectly with other hub genes (Figure 6C). For instance, mir-149-3p targeted only *METTL14*, whereas mir-1-3p targeted both *YTHDC1* and *YTHDF2*. In terms of transcriptional regulation, the 10 hub genes interacted with several TFs involved in important biological functions, including embryonic development-related genes (*FOXC1, FOXL1, FOXA1, GATA2, KLF5, TF2P2A,* and *NR2F1*), cell cycle-related genes (*E2F1, YY1, TP53, SP1,* and *E2F4*), Wnt pathway-related genes (*HNF4A, NFYA, TEAD1,* and *PAX2*), immune response-related genes (*STAT3, STAT1, FOS, RUNX2, CREB1, FOX O 3,* and *GATA3*), and gluconeogenesis-related genes (*CEBPB, SREBF1, NRF1,* and *PPARG*) (Figure 6D). Taken together, these results indicate that the hub genes are associated with specific miRNAs and TFs, but also share common miRNAs and TFs, suggesting that they may be involved in the same regulatory processes and thus reflect similar biological functions. The crosstalk between hub genes and miRNAs or TFs may play an important role in the occurrence of RPL by regulating multiple biological functions.

### Correlation features of hub genes

Hub genes tend to come from the same family and therefore may be closely linked or may share significant correlations. To test this, we analyzed pairwise correlations between all 10 hub genes. (Supplementary Figure 2A, B). In the correlation bubble plot, the size of the bubble represents the size of the data. The results showed that most genes were positively correlated, although a few were negatively correlated. The most significantly positively correlated gene pair was *METTL14-YTHDF2* and the most significantly negatively correlated gene pair was *METTL3-METTL14*.

To further investigate the strength of the relationship, scatter plots of correlation coefficients were generated for the gene pairs (Supplementary Figures S2C, D). The correlation coefficients of *METTL3-METTL14* and *METTL14-YTHDF2* were -0.5152 (*p* < 0.01) and 0.5983 (*p* < 0.001), respectively. Overall, the results showed that the hub genes were closely correlated.

### Unsupervised clustering based on hub genes

**FIGURE 7:**
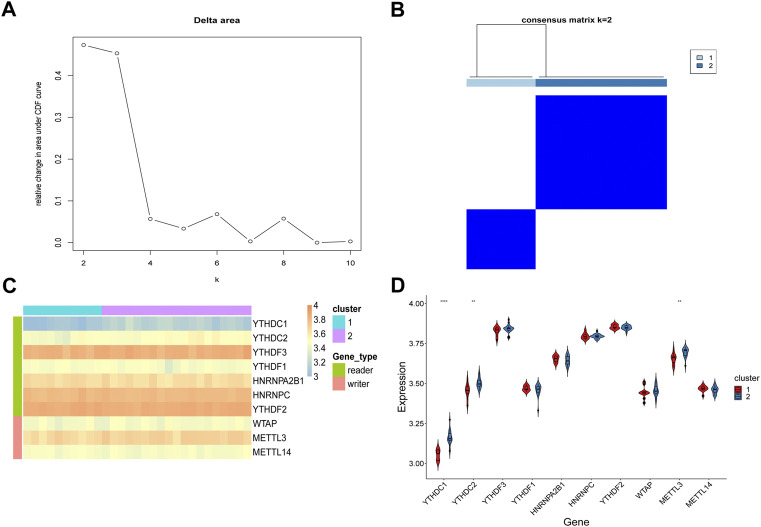
Unsupervised cluster analysis of hub genes in RPL samples. **(A)** Elbow plot. Relative changes in the area under cumulative distribution function curve. **(B)** Consensus matrix heatmap. Identification of two distinct RPL clusters. **(C)** Heatmap of unsupervised clustering of 10 hub genes in the two clusters. **(D)** Expression status of the 10 hub genes in the two RPL clusters. **p < 0.01; ****p < 0.0001. RPL, recurrent pregnancy loss.

Due to the heterogeneity among patients, the inter-individual heterogeneity may have adverse effects on the clinical therapy. Unsupervised clustering of samples based on hub genes will help distinguish samples with different disease statuses and reclassify the samples. Therefore, we performed unsupervised consensus cluster analysis of the RPL samples based on the expression data of the 10 hub genes. When two clusters were predicted, the consistency matrix heatmap showed a clear distribution of samples and the scatter at the corresponding position of the elbow plot was at the highest position. Thus, we chose k = 2 as the optimal number of clusters for unsupervised clustering and clustered all samples into two clusters, with 10 samples in Cluster-1 and 19 samples in Cluster-2 (Figures 7A,B). We then compared the expression levels of the 10 hub genes in samples in the two clusters (Figures 7C,D). As shown in Figure 7D, significant differences in the expression levels of three of the 10 hub genes were observed between samples in the two clusters: *YTHDC1* (*p* < 0.0001), *YTHDC2* (*p* < 0.01), and *METTL3* (*p* < 0.01), indicating that these three genes may be important distinguishing factors and may reflect the validity and accuracy of the clustering results. *YTHDC1* and *YTHDC2* which belong to the same family, showed extremely high variability in expression levels (*p* < 0.01), indicating that genes in the *YTHDC* family differ significantly among RPL cases. This observation warrants further investigation.

### Biological characteristics of the two RPL subtypes

**FIGURE 8:**
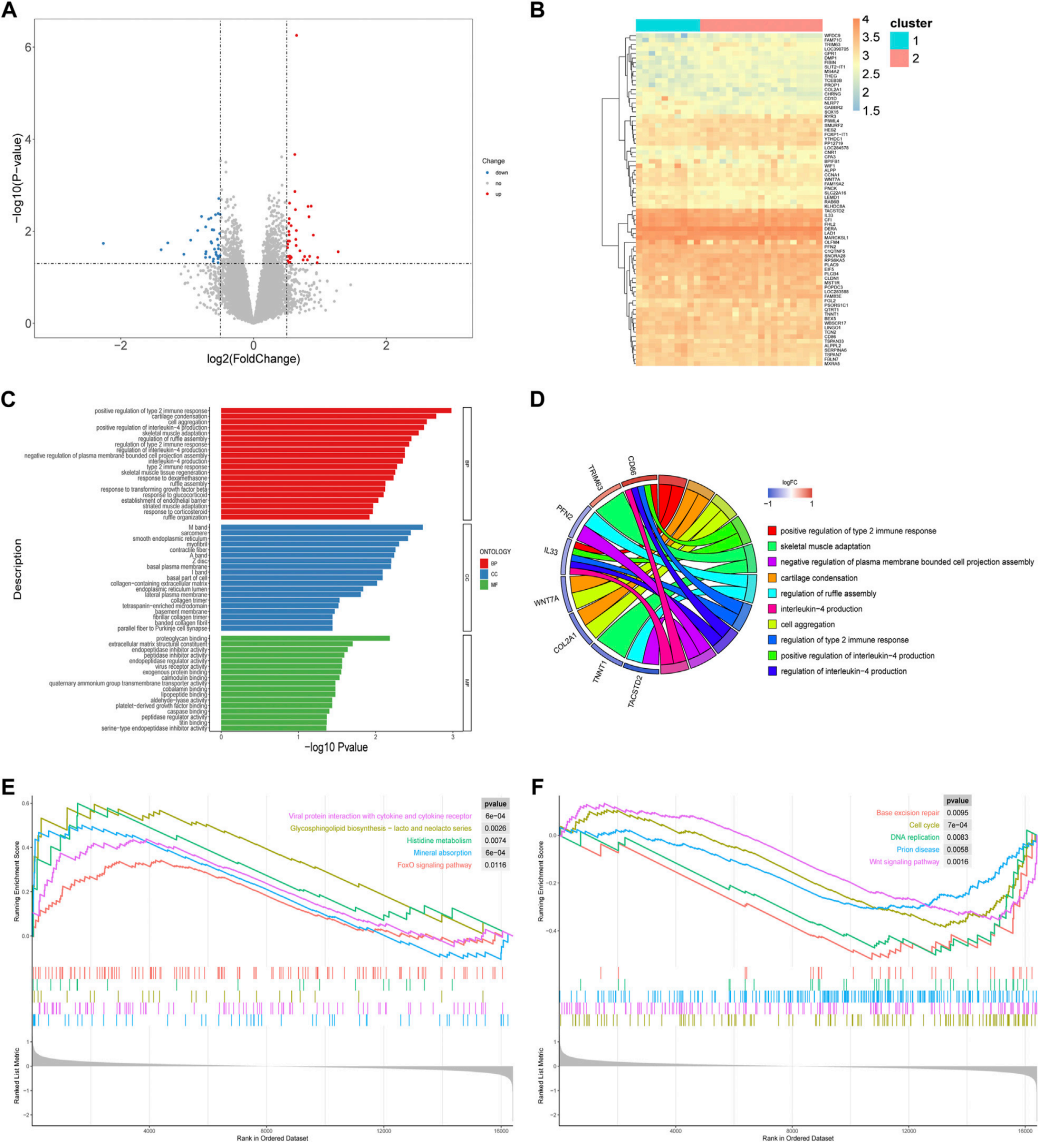
Differences in biological characteristics of two RPL subtypes. **(A)** Volcano plot showing the distribution of genes that were differentially expressed between the two RPL clusters. **(B)** Heatmap of DEGs. **(C)** GO enrichment analysis. The 20 most significant GO terms in of BP, CC, and MF are shown. **(D)** Chord plot of GO enrichment analysis. The top 10 GO terms, marked in different colors, are located in the right semicircle, and correlated genes are located in the left semicircle. **(E)** The top five upregulated pathways that were enriched, based on GSEA. **(F)** The top five downregulated pathways that are enriched, based on GSEA. DEGs, differentially expressed genes; GO, Gene Ontology; BP, biological process; CC, cellular component; MF, molecular function; GSEA, gene set enrichment analysis; RPL, recurrent pregnancy loss.

Differential gene expression analysis was used to determine the biological characteristics of the two RPL subtypes. A total of 74 DEGs were identified (Figures 8A,B). We conducted GO analysis and GSEA to further understand the molecular mechanisms underlying the DEGs involved in the m6A-mediated regulation of the two RPL subtypes.

A total of 191 GO terms were identified using the GO enrichment analysis (Supplementary Table S2), mainly related to the regulation of immune processes and tissue formation. The top five items based on the enrichment scores were positive regulation of type 2 immune response, positive regulation of interleukin (IL)-4 production, cartilage condensation, cell aggregation, and skeletal muscle adaptation (Figure 8C, D).

GSEA identified a total of 38 enriched pathways, including the cell cycle (normalized enrichment score, NES = -1.626, *p* < 0.001), Wnt signaling pathway (NES = -1.557, *p* < 0.01), FoxO signaling pathway (NES = 1.436, *p* < 0.05), viral protein interaction with cytokines (NES = 1.724, *p* < 0.001), and glycosphingolipid biosynthesis-lacto and neolacto series (NES = 1.857, *p* < 0.01) (Figures 8E, F; Supplementary Table S3). Thus, the pathogenesis regulated by m6A methylation in Cluster-1 and Cluster-2 differs in both immune processes and tissue formation.

### Infiltration characteristics of the immune microenvironment in the two RPL subtypes

**FIGURE 9:**
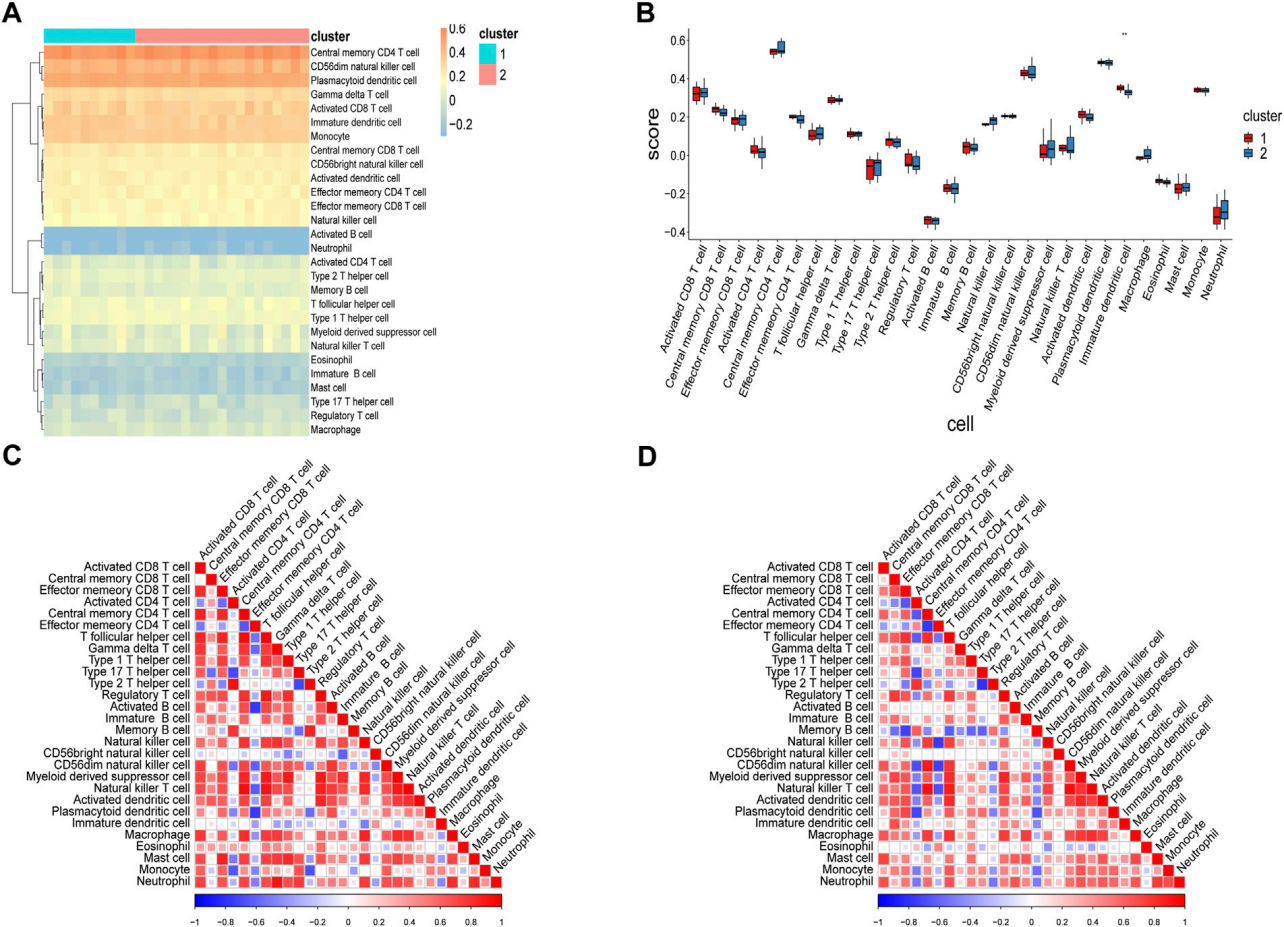
Analysis of immunocyte infiltration levels in two RPL subtypes using ssGSEA. **(A)** Heatmap of immune scores. Rows represent 28 immunocytes and columns represent samples. Orange represents a high level of infiltration, whereas blue represents a low level of infiltration. **(B)** Box plot of immune scores. The *x*-axis represents the 28 immunocytes, and the *y*-axis represents the level of immune infiltration, with each color representing a cluster. **(C)** Heatmap showing correlations between 28 immunocytes in Cluster-1. **(D)** Heatmap showing correlations between 28 immunocytes in Cluster-2. **p < 0.01. RPL, recurrent pregnancy loss; ssGSEA, single-sample gene set enrichment analysis.

Considering the significant differences in immune processes between the two subtypes identified using enrichment analyses, we analyzed differences in immune infiltration levels in the two RPL subtypes. Scores for 28 immunocytes in Cluster-1 and Cluster-2 were computed using ssGSEA (Figures 9A,B). The infiltration of immature dendritic cells (iDCs) was significantly higher in Cluster-1 than in Cluster-2 (*p* < 0.01).

**FIGURE 10:**
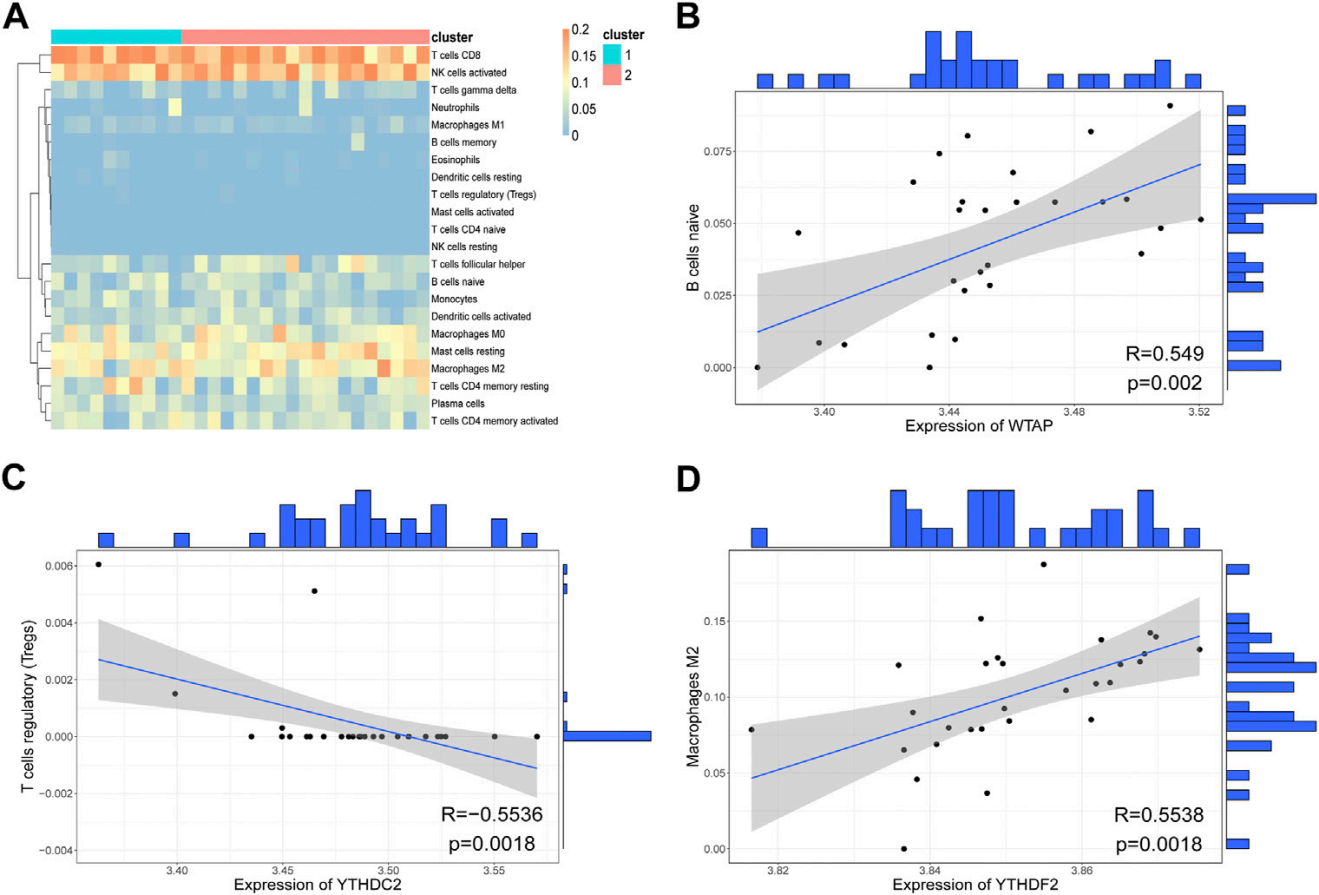
Evaluation of immunocyte infiltration levels using CIBERSORT. **(A)** Heatmap of immune scores. The rows represent 22 immunocytes, and the columns represent samples. **(B)** Scatter plot of the correlation between *WTAP* expression and the number of naive B cells. Each point in the graph represents a sample, the straight line is the correlation fitting curve, with the shaded region representing the confidence interval. The outer part of the graph is the histogram. **(C)** Scatter plot of the correlation between *YTHDC2* expression and the number of regulatory T cells (Tregs). **(D)** Scatter plot of the correlation between *YTHDF2* expression and the number of M2 macrophages.

Correlations between immunocytes in the two clusters were also calculated. As shown in Figures 9C,D, most immunocyte pairs were positively correlated, and only a minority were negatively correlated. Importantly, very few immune cell pairs were inversely correlated in the two clusters. For instance, iDCs and neutrophils were negatively correlated in Cluster-1 but positively correlated in Cluster-2, indicating differences in the immune microenvironments of samples in the two clusters. Analysis of immune infiltration levels of 22 immunocytes in the two clusters using CIBERSORT gave results similar to those obtained using ssGSEA (Figure 10A). Subsequently, correlations between hub genes and immunocyte abundance were calculated. The results revealed positive correlations between *WTAP* expression levels and the number of naive B cells (*r* = 0.5490, *p* < 0.01) and *YTHDF2* expression levels and the number of M2 macrophages (*r* = 0.5538, *p* < 0.01), but negative correlation between *YTHDC2* expression levels and the number of regulatory T cells (Tregs; *r* = -0.5536, *p* < 0.01) (Figure 10B–D). These results indicate that certain m6A regulators (*WTAP, YTHDF2* and *YTHDC2*) affect the infiltration levels of certain immunocytes (naive B cells, M2 macrophages, and Tregs) during RPL.

## Discussion

We identified 18 m6A regulators that were differentially expressed in RPL. We selected 15 of these and used them to construct a diagnostic model that could be used distinguish between RPL and control samples. We also demonstrated that the crosstalk between hub genes and miRNAs or TFs constituted the molecular regulatory network for RPL. We identified two RPL subtypes with significantly different biological processes and immune statuses based on novel signatures of the hub genes. To the best of our knowledge, this is the first comprehensive bioinformatic analysis of the effect of m6A modifications in the occurrence of RPL.

First, we integrated the expression profiles of GSE165004 and GSE26787 datasets from the GEO database and found that the expression of 18 m6A regulators was significantly altered in individuals with RPL, suggesting the involvement of m6A regulators in the occurrence of RPL. For example, expression of the m6A-modified eraser *ALKBH5* and writer *METTL3* were significantly upregulated in RPL samples, consistent with previous findings (Li et al., 2019; Qiu et al., 2021). However, *METTL14-ZC3H13* and *METTL3-CBLL1* were positively correlated, probably due to the fact that they regulate the same biological processes (Gong et al., 2020; Jiang et al., 2021) and therefore have a synergistic effect on gene expression. Taken together, these results suggest that the occurrence of RPL is associated with abnormal expression of and crosstalk between these 18 m6A regulators.

Next, we constructed an RPL diagnostic model using 15 m6A regulators that could distinguish between control and RPL samples based on risk scores. Some of these regulators have previously been associated with RPL or spontaneous miscarriage. *HNRNPC* overexpression reportedly causes abnormal expression of the paternal gene in RPL (Jena et al., 2021). Mutations in *FMR1* have been linked with RPL, and additional screening for CGG repeat amplification mutations in *FMR1* has been recommended for women with a history of spontaneous miscarriage (Dean et al., 2019; Ma et al., 2019; Blyth et al., 2021). Inhibition of *METTL14* expression reduces viability, proliferation, and migration of HTR8 cells, and may serve as a potential novel target for diagnosis and treatment of spontaneous miscarriage (Qin et al., 2020). In this study, *CBLL1, RBM15, FTO, YTHDC2, IGF2BP2, PRRC2A, eIF3B, eIF3H, LRPPRC, TRMT112,* and *XRN1* were reported to be associated with the occurrence of RPL for the first time.

For prognostic biomarkers, time-dependent ROC reveals both disease status and factor values change over time (Bian et al., 2022). In current study, 15 m6A regulators, especially four regulators with the highest absolute influence coefficient values (*YTHDF2, METTL14, FMR1* and *CBLL1*) were considered as predictive markers, constructing a diagnostic model for RPL that had good predictive efficiency and robustness in differentiating RPL from control samples. Due to the heterogeneity of RPL and the lack of clinical data, we were unable to evaluate the associations between risk indicators and disease status of RPL patients. However, we also validated the predictive efficacy of the diagnostic model using various methods and confirmed that it exhibited excellent predictive power. The recall curve showed that the diagnostic model had an overall AUC value of 0.994, demonstrating excellent diagnostic predictive power. The area under the ROC curve value of *YTHDF2*, the gene with the largest absolute coefficient, was 0.69. The DCA curve showed that the model yielded additional benefits over all interveners and non-interveners, indicating a good clinical effect. To the best of our knowledge, this is the first RPL diagnostic model based on m6A regulators.

Expression levels of genes that regulate the same biological processes are generally highly correlated. We identified 10 hub genes that were correlated. As shown using predicted regulatory networks, mir-376c-3p, mir-421, and mir-139-5p directly targeted *YTHDF1*, consistent with previous reports (Zheng et al., 2020; Zhou et al., 2020; Chi et al., 2021). *FOXA1* and *GATA2*, embryonic development-related TFs, were upregulated in RPL villi and promoted trophoblast migration and apoptosis (Du et al., 2020; Luan et al., 2020)*.* We predicted that *FOXA1* and *GATA2* could regulate *YTHDF2*, suggesting that trophoblast dysfunction may be influenced by m6A regulatory processes. We also identified multiple miRNAs and TFs that targeted the hub genes, suggesting that the mechanism underlying RPL occurrence involves complex regulatory networks that may be associated with the heterogeneity of patients with RPL, and further experiments are needed to identify specific regulatory mechanisms.

Despite the heterogeneity among patients with RPL, we identified two distinct molecular RPL clusters using unsupervised cluster analysis based on the expression data of 10 hub genes. The two clusters differed significantly in gene expression levels, immune responses, and tissue formation processes. For example, compared with Cluster-2, Cluster-1 was significantly more enriched in the positive regulation of type 2 immune responses and positive regulation of IL-4 production. Successful pregnancy is closely associated with transition from type 1 to type 2 immune responses (Zhao et al., 2021) and IL-4 cytokine is a signature of type 2 immunity (Gause et al., 2020). Furthermore, GSEA showed that the FoxO signaling pathway was activated in Cluster-1, while the cell cycle and Wnt pathways were inhibited. Previous studies have shown that Wnt and FoxO signaling pathways are associated with trophoblast cell function and embryonic development (Muñoz-Espín et al., 2013; Harrison et al., 2017; Li et al., 2017) and were identified as high-risk pathways in women who experienced spontaneous miscarriages (Cui et al., 2021). This is the first study to classify RPL based on m6A regulators, identifying two subtypes with distinct mechanisms of pathogenesis that differ both in immune responses and tissue formation-related signaling pathways.

We conducted immune cell infiltration analysis to further analyze differences in the immune microenvironments in the two RPL subtypes. We found that samples in Cluster-1 had higher immune infiltration levels of iDCs. Dendritic cells (DCs) are the most powerful antigen-presenting cells, capable of suppressing the maternal immune rejection of semi-allogeneic embryos (Audiger et al., 2017). iDCs, which are derived from DC precursors, are predominantly expressed in the decidua during early gestation and induce immune tolerance at the maternal–fetal interface during the peri-implantation period. Studies have suggested that the immunological mechanism underlying RPL may involve decidual iDCs that are stimulated to differentiate and develop into mature DCs following exposure to inflammatory factors, which further activates the proliferation of naive T cells, breaks the Th1/Th2 cell balance, and leads to rejection and abortion (Qian et al., 2015). Thus, we hypothesize that iDCs play a vital role in inducing RPL in Cluster-1, which is more likely to exhibit inflammation at the maternal–fetal interface. Furthermore, M2 macrophages were positively correlated with *YTHDF2* in both RPL subtypes, consistent with the involvement of *YTHDF2* in macrophage activation (Gu et al., 2020). The predominance of decidua M2 macrophages is an important contributor to maternal–fetal tolerance during early pregnancy (Zhang et al., 2019). Further analyses of *YTHDF2* are needed, given its high diagnostic value in RPL.

To the best of our knowledge, this is the first study to systematically analyze the relationship between m6A regulators and RPL from genomic, proteomic, and immunomic perspectives, and provides novel insights into RPL occurrence. The model we have developed has potential application in early RPL diagnosis. This study also provides an in-depth understanding of regulatory mechanisms underlying RPL immune microenvironments and serves as a basis for stratification and refined management of RPL subtypes in clinical practice.

This study had several limitations. First, the study was based on bioinformatics analysis only, and additional *in vitro* and *in vivo* experiments are required to validate the findings. Second, an extensive clinical cohort with a larger sample size and more complete clinical data is needed to validate the predictive value of our diagnostic model for RPL, which requires a long period of observation. We hope to conduct case-control and laboratory studies to validate our findings.

We highlight several areas in which further work is needed to deepen our understanding. First, we will perform histological validation in villus samples of RPL to examine basic expression of m6A regulators with highly diagnostic value, such as *YTHDF2, METTL14, FMR1* and *CBLL1* by Western blot, real time PCR, immunohistochemistry, immunofluorescence assays, etc. Second, to clarify the function of m6A regulators and hub genes in two RPL clusters, loss-of-function and gain-on-function studies with tissue-type specificity and cell-type specificity remain warranted. Third, the co-expression and interaction among hub genes is a new exciting frontier that awaits further investigation. Co-Immunoprecipitation and pull-down assays would suggest powerful evidence for molecular mechanisms in the pathogenesis of RPL. Moreover, we would like to establish an RPL cohort and follow up the eligible individuals to validate the predictive efficiency of the diagnostic model.

## Conclusion

We showed that m6A modification plays a critical role in the occurrence of RPL, and m6A regulators are highly correlated at the transcriptomic level. A diagnostic model that can distinguish between RPL and control samples was constructed using risk scores for 15 m6A regulators. The crosstalk between the hub genes and miRNAs or TFs was used to construct a regulatory network illustrating the regulation of biological mechanisms underlying RPL. Based on distinct hub gene signatures, two RPL subtypes with significantly different biological processes and immune statuses were identified, increasing our understanding of the heterogeneity of the RPL population and providing scientific evidence for personalized diagnosis and treatment of RPL.

## Data Availability

Publicly available datasets were analyzed in this study. The names of the repository/repositories and accession number(s) can be found in the article Supplementary Material.

## Abbreviations

LASSO, least absolute shrinkage and selection operator; m6A, N6-methyladenosine; RPL, recurrent pregnancy loss; PPI, protein–protein interaction; GSEA, gene set enrichment analysis; GO, gene ontology; TFs, transcription factors; ssGSEA, single-sample gene set enrichment analysis; BP, biological process; CC, cellular component; MF, molecular function; iDCs, immature dendritic cells; DCA, decision curve analysis; ROC, receiver operator characteristics; AUC, area under the curve; DEGs, differentially expressed genes.
