# Supplementary material for: Molecular subtype identification and predictive power of N6-methyladenosine regulator in unexplained recurrent pregnancy loss
Source: Front Genet. 2022 Sep 2;13:925652. doi: 10.3389/fgene.2022.925652 (PMC9478558; doi:10.3389/fgene.2022.925652)
Supplement: Supplementary file 4 [file DataSheet1.DOCX]

Supplementary Material


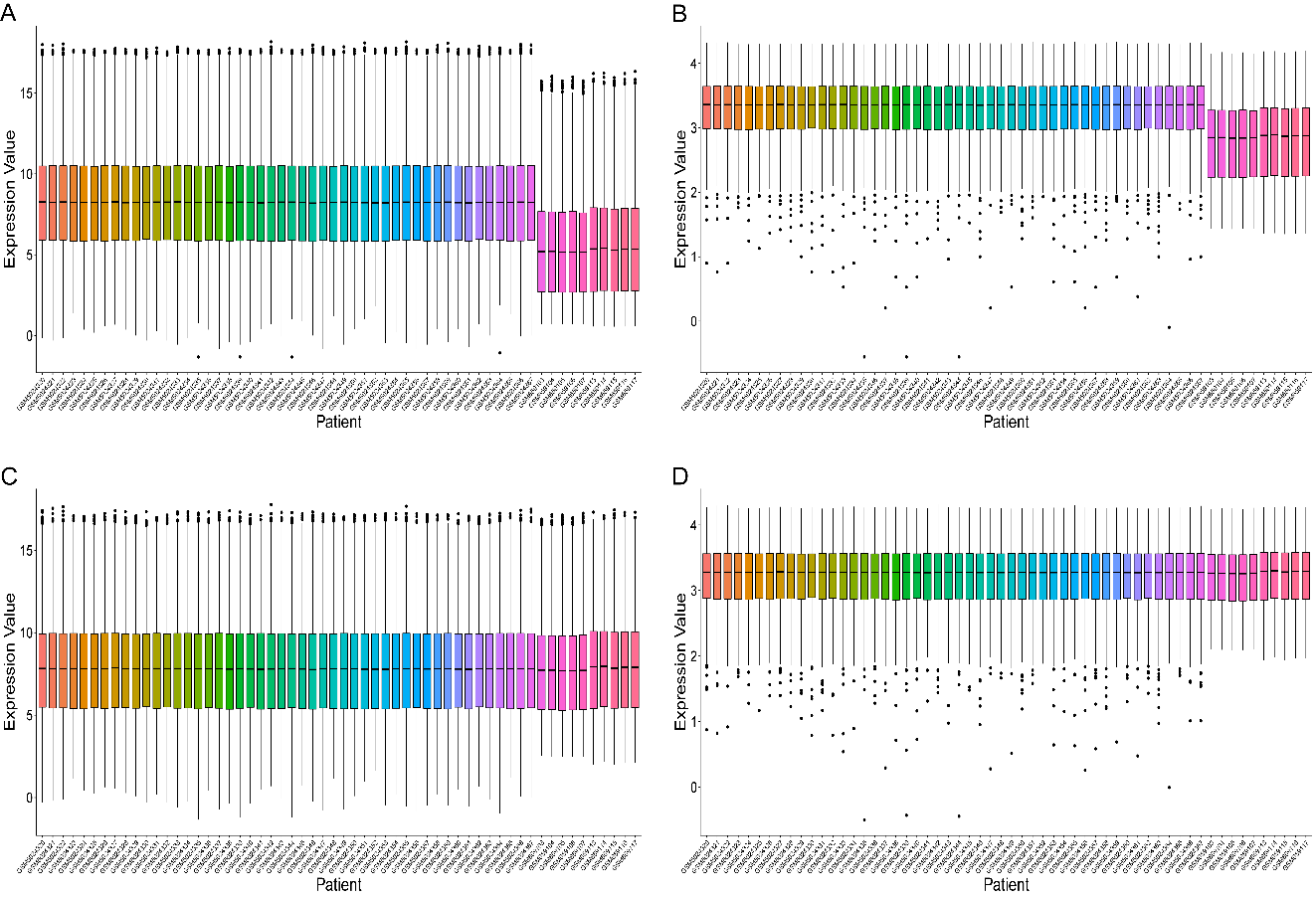


**Supplementary Figure 1. Expression distribution and batch effect correction for all samples.** (**A**) Expression distribution without log standardization and batch effect correction for all samples. (**B**) Expression distribution with log standardization and without batch effect correction. (**C**) Expression distribution with batch effect correction and without log standardization. (**D**) Expression distribution with batch effect correction and log standardization.

**
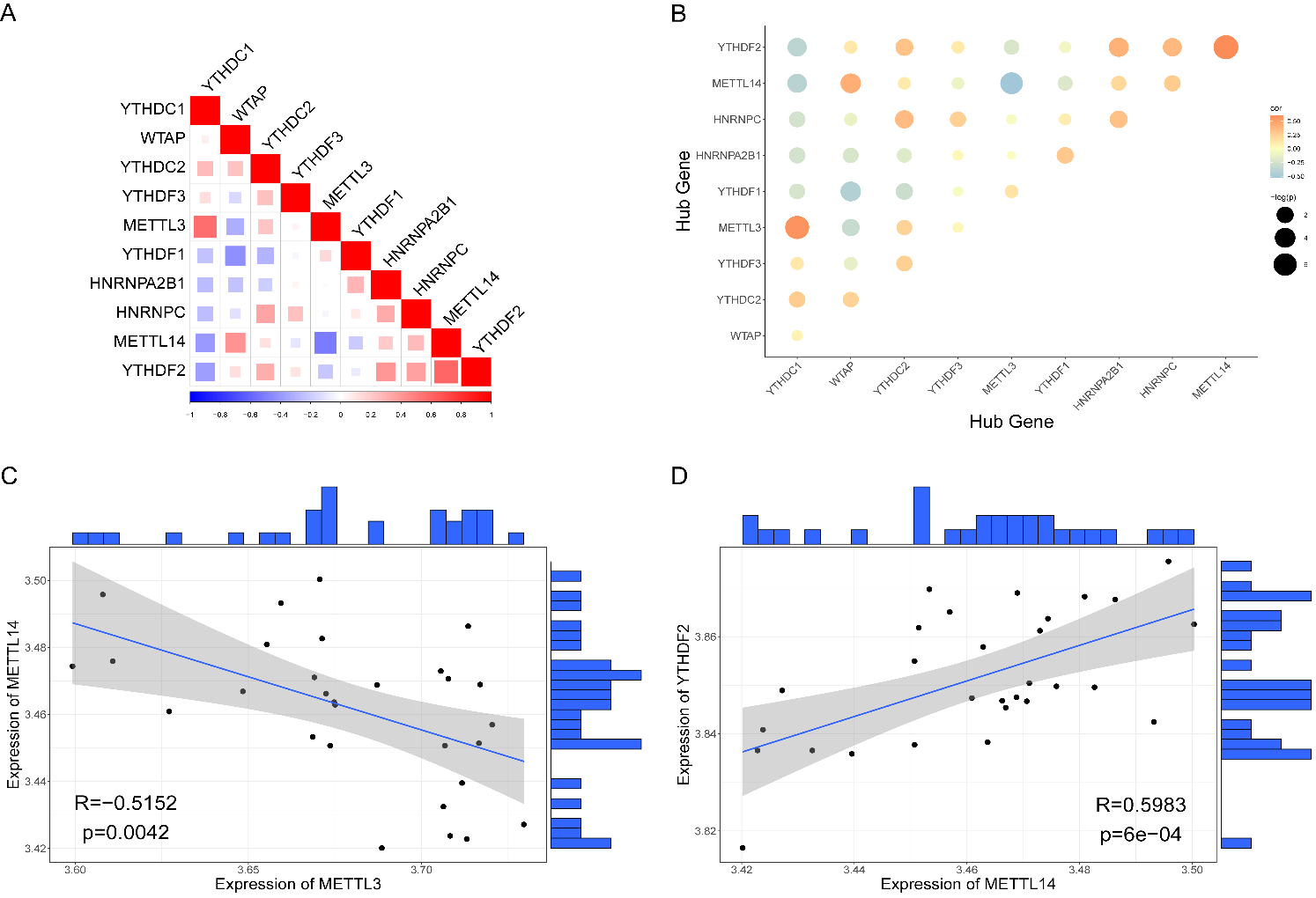
**

**Supplementary Figure 2. Correlation analysis of hub genes.** (**A**) Correlation heatmap among hub genes. (**B**) Correlation bubble plot among hub genes. (**C**) Correlation scatterplot of *METTL3* and *METTL14*. (**D**) Correlation scatterplot of *METTL14* and *YTHDF2*.
